# Fatal Poisoning by Nitrate of Potassa

**Published:** 1855-04

**Authors:** John Snowden


*Fatal Poisoning by Nitrate of Potassa.* By JOHN SNOWDEN, M.D. —A German, who spoke English imperfectly, went into a store, and asked for " bitter salt," meaning sulphate of magnesia. The attendant supposed he meant saltpetre, and gave him half-a-pound. The man took three ounces and a half at one dose. His bowels were opened three times within three or four hours. He complained of a slight sense of heat in the epigastrium, and drank a good deal of water. About five hours after having taken the saltpetre, he suddenly fell out of his chair and died.

The marked peculiarity, in this case, is the absence of the painful symptoms which usually follow the ingestion of irritant poisons; and the question arises, how was death produced? Certainly not by inflammation of the stomach, for he complained of nothing but a slight sense of heat in the stomach. The poison must have acted by destroying the vitality of the blood. There was no *post-mortem* examination. The rigor mortis was very imperfect, the lips of almost a natural pink hue, and the appearance of the countenance so life-like, that some persons who were present doubted the propriety of interment on the third day. —*New Jersey Medical Reporter.*

